# Demographic change and selection patterns in thoroughbred racing horses over the 20^th^ century

**DOI:** 10.1016/j.isci.2026.116830

**Published:** 2026-07-17

**Authors:** Hojjat Asadollahpour Nanaei, Andaine Seguin-Orlando, Kerry Negara, James N. MacLeod, Ted Kalbfleish, Ludovic Orlando

**Affiliations:** 1 Centre d’Anthropobiologie et de Génomique de Toulouse, CNRS/University of Toulouse, 37 Allées Jules Guesde, Toulouse 31000, France; 2 Negara Film TV Media, Melbourne, VIC, Australia; 3 Gluck Equine Research Center, Department of Veterinary Science, University of Kentucky, Lexington, KY, USA

**Keywords:** ancient DNA, thoroughbreds, demography, inbreeding, selection, racing industry

## Abstract

The Thoroughbred horse is a legacy of centuries of selective breeding for speed, emerging from the admixture of British mares and imported Oriental stallions. While previous work explored its genetic diversity, inbreeding, and selection signatures, the breed demographic and selection dynamics during the 20^th^-century remain poorly understood. Here, we sequenced the genome of Phar Lap, a late-1920s/early-1930s racing champion, and analyzed the largest time-series of Thoroughbred whole-genome sequences, within a global panel of 850 horse genomes. Demographic modeling reveals a marked decline in genetic diversity since 1932, driven by a reduced effective population size, particularly until 1960. This aligns with a modest yet significant increase in long runs-of-homozygosity (ROH), suggesting that breeding practices only partially mitigated ROH accumulation and inbreeding depression. Temporal shifts in genetic variation identify candidate loci linked to racing performance, offering insights into past and ongoing selection, and new loci of major potential for the global racing industry.

## Highlights

• Genome sequence of Phar Lap, a racetrack champion deceased in 1932
• Time-stamped phased SNP panel of 172 Thoroughbreds from 1905 to the present-day
• Low genetic diversity associated with a sharp demographic decline between 1929 and 1965
• Consanguinity increased since the 1960s, with dynamic selection targets after the 1930s

## Graphical abstract

**Figure:**
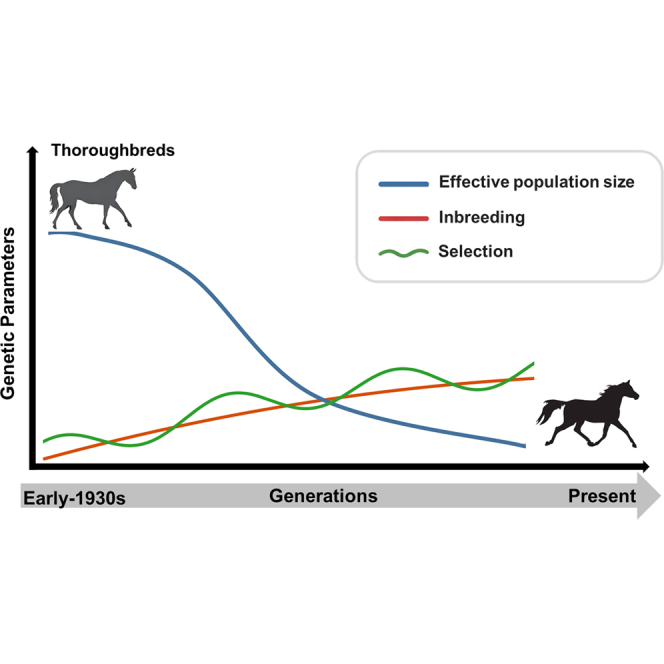

## Teaser

Equine genetics; Biological sciences; Genetics

## Introduction

For millennia, horses have played a central role in human history, serving mobility and warfare. While they remain critical to developing countries, their role is restricted to leisure and sport in Western societies.^1^ The athletic performance of horses on the racetrack is celebrated around the world, with the Thoroughbred representing one of the most popular and fastest breeds.^2^^,^^3^ The genetic origins of the Thoroughbred can be traced to imported Oriental stallions that were mixed with local British mares by the late 17^th^ and early 18^th^ centuries.^4^^,^^5^ Once the Studbook was established in 1791, the Thoroughbred population remained effectively closed to outside genetic input. As a result, more than 80% of its gene pool can be traced back to only 31 founding horses, limiting genetic diversity despite a now large global population size (*N* ∼ 500,000).^5^^,^^6^ Selectively bred for superior athletic ability, their influence extends worldwide, and was foundational to many light horse breeds.^2^^,^^7^

Previous genomic research in Thoroughbreds has explored genetic diversity,^6^^,^^8^ inbreeding,^6^^,^^9^^,^^10^^,^^11^ and selection signatures,^6^^,^^12^^,^^13^ with a primary focus on uncovering the genetic basis of their extraordinary racing performance.^2^ For example, genetic variation at the *MSTN* locus strongly impacts speed at short distances, with a 227 bp SINE insertion segregating at high frequencies among horses that require speed in sprint races.^14^^,^^15^^,^^16^ More recent work based on high-density SNP panels^6^ or whole genome sequence data^8^ has also investigated the genetic impact of breeding practices in Thoroughbreds, revealing pervasive inbreeding and relatively limited deleterious load.^9^ However, only one historical Thoroughbred genome has been sequenced so far,^17^ leaving the breed’s genetic pool unsampled between 1905 and the modern period. Yet, museum collections comprise biological remains of many historical racing champions. With continuously improving genomic tools to retrieve ancient DNA,^18^ the long-term variation of Thoroughbred genetic diversity can be characterized.

In this study, we leveraged ancient DNA techniques to sequence the genome of Phar Lap, one of Australia’s most legendary racehorses of the early 20^th^ century. Rising to prominence during the Great Depression, his enduring legacy extends beyond the racetrack to the present day, decades after his death in 1932.^19^ By combining Phar Lap’s genome with the largest time-resolved panel of Thoroughbred whole-genome sequences assembled to date, we reconstruct temporal patterns of genetic diversity, genomic inbreeding, and effective population size throughout the recent evolutionary history of the breed. We further examine shifts in selective pressure by identifying candidate genomic regions showing temporary-dynamic genetic patterns linked to racing performance and athletic traits. Collectively, our analyses offer a genome-wide perspective on how prolonged artificial selection and demographic constraints have shaped the history of Thoroughbreds, thereby highlighting the broader value of genomic time-series for tracking the evolution of economically and culturally significant domestic animal lineages.

## Results and discussion

### Genome panel

We collected an envelope containing 6 tail hair samples from Phar Lap, the iconic Australian racetrack champion who was born in 1926 but died suddenly in 1932 following suspected poisoning.^20^^,^^21^ The material was sealed and authenticated by Tommy Woodcock, who served as Phar Lap’s strapper and primary caretaker, and has since remained in the family of one of the co-authors (K.N.). Using dedicated ancient DNA facilities, we sequenced Phar Lap’s genome (PHAR) to an average depth-of-coverage of 9.91-fold from 12 triple-indexed DNA libraries and 201.7 million Illumina reads (including 191.0 million collapsed read pairs).

To contextualize PHAR within the broader genetic diversity of Thoroughbreds, we integrated sequences for 328 additional Thoroughbred genomes (THRB) (Table S1), characterized at an average 21.30-fold depth-of-coverage (median = 16.91-fold). Among these, 170 individuals have documented birth dates spanning five decades (1965–2020) (Table S2), with >97% originating from North America.^8^ We further combined previously published genomic data for two early 20th-century horses, Alfort (ALFT), born 1882, deceased 1903,^22^ and; Dark Ronald (DRKN), born 1905, deceased 1928,^17^ alongside 522 representatives of other breeds or populations to expand temporal and geographic breadth (Table S3). ALFT is a historical Irish horse preserved at the École Nationale Vétérinaire de Maison Alfort (ENVA, France).^22^ DRKN is an English Thoroughbred stallion preserved in the Halle museum in Germany. He sired numerous Thoroughbred and sport horses, leaving a long-lasting influence on both Thoroughbred and warmblood breeding.^17^

Following genotyping with Graphtyper^23^ and phasing with Beagle 5.2,^24^ we identified ∼16.6 million high-quality single nucleotide polymorphisms (SNPs), segregating at a minor allele frequency (MAF) of 5% among post-1965 genomes (*N* = 850) (Figure S1). This comprehensive SNP panel was used with GLIMPSE2^25^ to impute the genome-scale autosomal variation for the three low-to-moderately covered ancient genomes of the early 20^th^ century (1.01-fold–9.91-fold). To validate imputation reliability, we down-sampled a subset of ten high-quality THRBs to match the coverage of these ancient genomes (Table S4), performed imputation, and compared the imputed genotypes to those called using the full sequence data. This procedure indicated genotyping errors of 0.02%–0.31% for PHAR and 0.02%–1.43% for the other two (Figures S2A–S2C), with imputation accuracies (defined as the correlation level between imputed and true genotypes) ranging from 97.5% to 99.1% at MAF = 5% (Figures S2D–S2F).

### Population structure

Principal component analysis (PCA), neighbor-joining (NJ) phylogenetic inference, and ADMIXTURE^26^ ancestry profiling collectively confirmed the long-established genetic distinction between domesticated and Przewalski’s horses (Figures S3–S5). This differentiation aligns with their estimated ∼26 k years of divergence,^27^ and the archaeogenetic evidence for horse domestication dating to 4.2–4.6 k years ago.^22^^,^^28^

**Figure 1:**
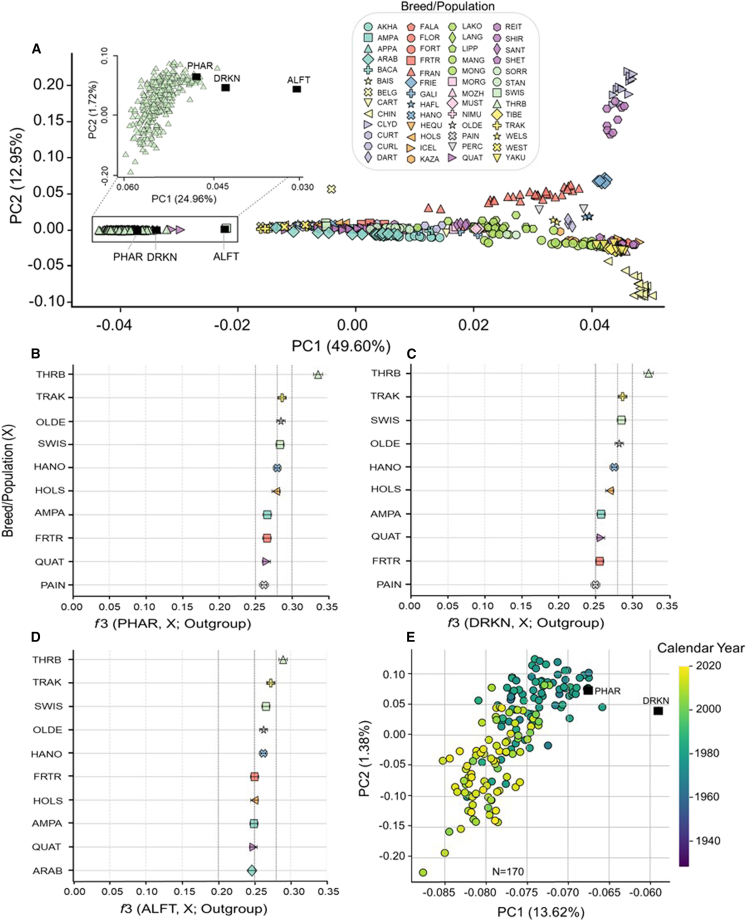
Population structure and genetic affinities (A) PCA of globally distributed modern horse breeds (*N* = 850) and three ancient specimens (PHAR, DRKN, and ALFT) (*N* = 2,642,046 bi-allelic unlinked SNPs, with MAF = 5%). The inlet shows an independent PCA, restricted to Thoroughbreds (*N* = 328) and the three ancient specimens. Percentages between parentheses indicate the fraction of the total genetic variance explained by the first and second principal components. (B–D) *f*3-outgroup statistics measuring genetic sharedness between three ancient samples (PHAR, DRKN, and ALFT) and modern horse breeds. A total of *N* = 23 Prewalski’s horses were selected as the outgroup. Higher *f*3-outgroup values indicate closer genetic affinities with the breeds or populations considered (X). The top-10 breeds or populations are shown (for a complete graph, see Figure S6). (E) PCA of modern (*N* = 170) and ancient (*N* = 2) Thoroughbreds. The analysis was restricted to those individuals with known birth dates, as reflected by the color gradient.

Excluding Przewalski’s horses to gain resolution into the population structure of domesticated horses, PHAR and DRKN were found to cluster along the first PC within THRB, while ALFT placed further away (Figure 1A), reflecting its NJ phylogenetic placement outside Thoroughbreds (Figure S4). *f*3-outgroup statistics clearly indicated that PHAR and DRKN were significantly closer to THRB than any other breed or population present in our dataset. Conversely, ALFT showed overlapping proximity with four other breeds of European origins (Trakhener, TRAK; Swiss Warmblood, SWIS; Oldenburg, OLDN, and; Hanoverian, HANO) (Figures 1B–1D and S6). The more distant genetic affinities between ALFT and THRB are consistent with historical records from the Maison-Alfort Veterinarian collection, describing the 1882 specimen simply as an “*Irish horse*.” This contrasts with the certified origins of DRKN and PHAR, both registered to the Thoroughbred studbook. We, thus, excluded ALFT from the downstream analyses aimed at characterizing the genetic history of Thoroughbreds in the 20^th^ century.

### Heterozygosity and inbreeding

**Figure 2:**
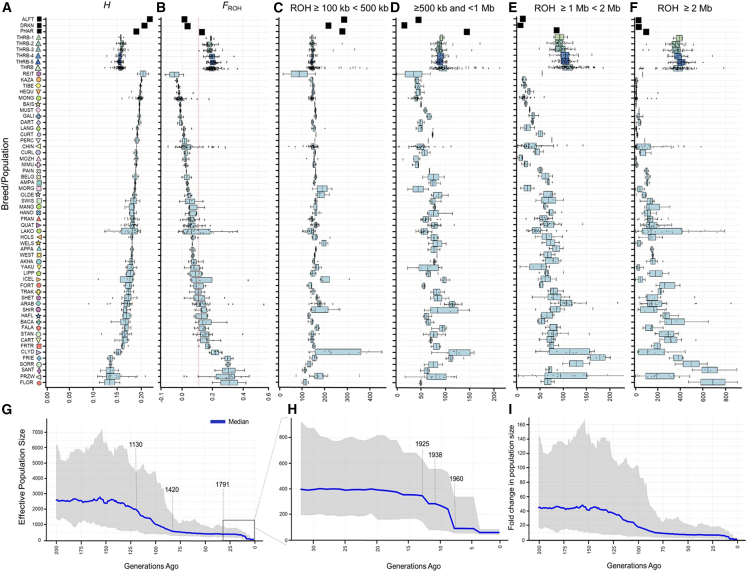
Genetic diversity, inbreeding, ROH, and demographic trajectory (A) Autosomal heterozygosity (*N* = 2,642,046 unlinked SNPs). (B) Inbreeding coefficient (*F*_ROH_) across breeds or populations. (C–F) The total length encompassing ROH (Mb) broken down in four size categories: short (≥100 kb and <500 kb), intermediate (≥500 kb and <1 Mb), long (≥1 Mb and <2 Mb), and very long (≥2 Mb). In (A–F), analyses were carried out across a diverse set of breeds and populations. For visualization clarity, only major populations are shown. Boxplots represent the 25%, 50%, and 75% quantiles, with upper and lower whiskers showing values within the 1.5 interquartile range. THRB-1: 1965–1969, THRB-2: 1970–1979, THRB-3: 1980–1989, THRB-4: 2000–2009, THRB-5: 2010–2020. Thoroughbred individuals (*N* = 158) without available year of birth information were classified as THRB. (G and H) Historical effective population size variation for the Thoroughbred population. Demographic changes were inferred using GONE.^29^ (I) Fold change in Thoroughbred population size. The figure shows the fractional change in Ne across generations for THBR horses. The solid blue line represents absolute median Ne values per generation (G and H), or scaled (I) relative to the year 2020 (generation 0). The shaded gray area indicates the 95% confidence interval (2.5^th^–97.5^th^ percentiles) across *N* = 100 replicates.

Restricting PCA to horses associated to known birth dates revealed strong temporal genetic differentiation within Thoroughbreds, with the first two PCs significantly correlated with time (Pearson correlation, r^2^ = 0.695 and 0.671, *p* value < 10^−23^), and PHAR and DRKN placed at one extreme of the distribution (Figure 1E and Table S2). Given the closed nature of the Thoroughbred studbook, and the separate position of Thoroughbreds in the global PCA (Figure 1A), these patterns likely reflect a combination of genetic drift and long-term changes in breeding practices affecting the breed, rather than admixture. Importantly, Thoroughbred autosomal heterozygosity appeared at the tail of the global distribution across breeds or populations, intermediate between Clydesdale (CLYD) and French Trotter (FRTR), and above breeds with a strong history of reported population bottlenecks, such as Sorraia (SORR), Santa-Cruz-Island (SANT) and Przewalski’s horses (PRZW) (Figure 2A). The limited heterozygosity levels measured here align with previous work, based on genome-scale SNP array genotyping,^6^^,^^30^ and whole-genome sequencing from a more limited number of breeds^9^ or Thoroughbred individuals.^11^

A marginal drop of autosomal heterozygosity was detected in the last five decades (THRB-1 to THRB-5; Kruskal-Wallis test, *p* value = 0.0112). Heterozygosity levels were, however, considerably higher in both PHAR and DRKN, comparable to those measured in Reit ponies and Asian breeds (Figure 2A and Table S5). This suggests a potential reduction in genetic diversity in the Thoroughbred population between the early 1930s and the mid-1960s, although the absence of genomes from this period prevents precise quantification of the change. Consistent with temporal patterns of heterozygosity, pairwise nucleotide diversity (π) remained relatively stable across the five decadal groups (THRB-1 to THRB-5), and generally lower than in several other horse breeds of Asian origins (Table S6).

Genomic inbreeding, as calculated by the total autosomal length of runs-of-homozygosity (*F*_ROH_), was relatively high compared to other breeds and populations and mirrored the global and temporal trends observed on heterozygosity. It significantly increased over the last five decades (Figure S7 and Table S7; Kruskal-Wallis test, *p* value <0.001), although marginally, and increased even further with respect to PHAR and DRKN (Figure 2B). The detected increase of inbreeding is consistent with the significant positive correlation reported by McGivney and colleagues^6^ over the last five decades for a considerably larger Thoroughbred panel (*N* = 10,118) between both individual *F*_ROH_ and per-year FIS coefficients on the one hand, and time on the other hand.

To explore this trend further, we binned ROHs into four size categories, following Silva and colleagues^31^: short (100 kb–500 kb), intermediate (500 kb–1 Mb), long (1–2 Mb), and very long (≥2 Mb) (Figures 2C–2F and Table S8). This classification enabled to disentangle whether genomic inbreeding resulted from enduring limited population sizes, or consanguinity (defined as mating between close-relatives), providing insights into the mechanisms underlying inbreeding depression.^31^^,^^32^

The number and total genomic length comprising short ROHs were largely comparable across the various modern breeds, including Thoroughbreds. However, THRB generally contained a larger fraction of long and very long ROHs, except relative to a few breeds, such as Arabian (ARAB) and CLYD, or SORR, Friesian (FRIE) and PRZW horses, known for the marked rise in consanguinity experienced after extreme demographic bottlenecks. Considering temporal trends, short ROHs only marginally declined over the last five decades (Figure 2C; Kruskal-Wallis test, *p* value = 0.0021), indicating that the breed effective size remained somewhat stable during the second half of the 20^th^ century. In contrast, the genome fraction of long and very-long ROHs significantly increased over the same time period (Figures 2E and 2F; Kruskal-Wallis test, *p* = 0.0017 and *p* = 0.0026, respectively). These results are consistent with the analyses from Bailey and colleagues who reported a significant rise in *F*_ROH_ for American Thoroughbreds born before (1965–1986) and after 2000 (2000–2020),^8^ considering all ROH larger than 300 kb. Similarly, using SNP array data of an extensive panel of 6,000 Thoroughbred horses born between 1995 and 2020, Hill et al. (2022)^10^ reported an increase in *F*_ROH_ over time in both European and Australian Thoroughbreds, regardless whether ROHs ≥5 Mb or shorter were considered. Combined, these analyses point to consanguinity, rather than major population declines, as a driver for the increasing genomic inbreeding trend detected since the mid-1960s. Interestingly, the genomic fraction encompassing ROHs of every size category was lower in DRKN than PHAR, suggesting that consanguinity was already increasing by 1928–1932, while population sizes declined.

### Demographic trajectory

Demographic modeling with GONE^29^ and using 57 Thoroughbreds born between 1965 and 2020 (Figures 2G and 2H, and Table S9), confirmed the modest effective size of the breed today. It also revealed a sharp declining trend during the ∼13 generations prior to 2020 (equivalent to ∼1925 assuming the average generation time of 7.4 years reported in Librado and colleagues^22^), corresponding to a median ∼6.04-fold reduction in effective population size (95% quantile range = 2.03– to 15.67-fold) (Figure 2I). Interestingly, our modeling also indicated that the ancestral stock giving rise to the Thoroughbred breed experienced a ∼3.49-fold (2.96- to 4.87-fold) demographic decline between 1130 and 1420 (Figures 2G–2I and S8). This may reflect changing breeding practices in late Middle Ages Europe, prior to the late 17^th^ century when horse racing became popular among the British gentry.^2^ Consistent demographic trajectories were obtained when using different THRB sets of individuals born before or after 2000 (Figure S8). We note that Thoroughbred effective sizes as estimated here (Ne = 43–83, median = 58) are considerably lower than those from McGivney and colleagues^6^ (Ne = 330 worldwide; Ne = 93–226 in subcontinental populations), who integrated a more limited number of SNPs (≤10,000) to retrieve point estimates for the present day, rather than fully reconstructing the explicit temporal trajectory.

Our analyses revealed that the history of Thoroughbreds was marked by a strong demographic collapse from the mid-1930s but a rather limited increase in genomic inbreeding mostly driven by inflating consanguinity since the 1960s. Given the limited number of historical genomes available, these patterns should be interpreted cautiously for the period prior to 1960, and viewed as indicative of a more extensive range of genetic variation, which should be documented at the population-level with additional historical genomes.

Importantly, Hill and colleagues^10^ demonstrated that ROH >5 Mb are associated with a reduced probability of Thoroughbreds entering into racing competitions. Our analysis further reveals a modest but significant temporal increase in the genome fraction comprising ROH >5 Mb across decadal Thoroughbred groups (Spearman ρ = 0.24, *p* = 0.0018; Figure S9). This trend extends the observations from Hill and colleagues for the period between 1995 and 2020, and suggests an overall decline in the likelihood of Thoroughbreds being selected for competition since the mid-1960s. This decline may have started even earlier, as the two historical genomes show markedly reduced ROH levels compared to those of their modern relatives (Figure S10). However, the magnitude of this temporal trend remains limited, indicating that breeding and selection practices have limited the accumulation of highly deleterious large-effect variants that could severely impair viability and racing participation. Long ROH are enriched for deleterious variants,^33^ and expose recessive harmful alleles to purifying selection.^8^^,^^17^ The efficacy of purifying selection is, however, reduced in populations of low effective size, where genetic drift may lead mildly deleterious small-effect variants to fixation. Previous work has established that the deleterious load in Thoroughbreds was limited.^9^ However, selection practices have not completely overcome the effects of genetic drift, given the evidence for inbreeding depression in racing and the prevalence of several performance-limiting heritable disorders in Thoroughbreds, such as recurrent exertional rhabdomyolysis,^34^ developmental orthopedic disease,^35^ and exercise induced pulmonary hemorrhage.^36^ It should also be noted that selection occurs at multiple stages in the Thoroughbred life cycle, with not even half of the foals entering training by the end of their third year, reflecting substantial early filtering prior to racing evaluation.^37^ While this pre-training attrition enhances the removal of severely disadvantaged individuals before reproduction, it has not been sufficient to fully prevent the persistence and cumulative effects of mildly deleterious alleles in a population with long-term low effective population size.

### Selection scans

To explore whether Thoroughbreds experienced significant shifts in breeding targets over time, we carried out two genome selection scans. In the first, we compared two sets of genomes representing the Thoroughbred population from the early 20^th^ century (PHAR and DRKN; *N* = 2) on the one hand, and modern Thoroughbreds on the other hand, formed by grouping individuals born after 1965 available in our dataset (*N* = 57) (Table S9). Importantly, the modern individuals used in this analysis predominantly originate from the United States (>97%), minimizing potential geographic bias among the analyzed groups. Although the number of early 20^th^ century genomes is limited to two, potentially limiting statistical power, they provide a temporal reference for allele frequency differences relative to modern Thoroughbreds.

**Figure 3:**
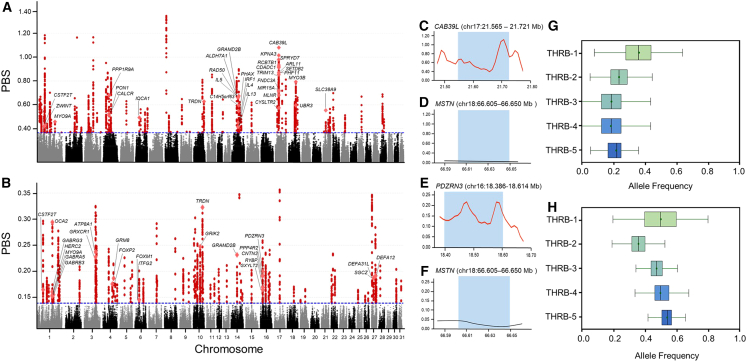
Selection scans (A and B) Manhattan plot of PBS estimated within 50 kb autosomal sliding windows, with a step-size of 10 kb. Each point represents a genomic window. The dashed horizontal line indicates arbitrary significance thresholds, reflecting the top-0.5% PBS values. Windows exceeding this threshold are highlighted in red as selective sweep candidates, except those containing genes of interest previously reported to have been positively selected in Thoroughbreds, shown with a pink diamond. In (A), PBS values were calculated between an ancient group of Thoroughbred individuals (PHAR and DRKN) and *N* = 57 modern Thoroughbreds. In (B), the analysis was repeated between those modern Thoroughbreds born before (*N* = 31) and after (*N* = 26) year 2000 (Table S9). (C–F) PBS profiles across genomic regions containing genes-of-interest previously reported to have undergone positive selection. The *CAB39L* and *PDZRN3* genes, but not *MSTN*, feature amongst exceptionally high-PBS regions. (G and H) Allele frequency trajectory at position rs397152648 (chr18:66,608,679) in the *MSTN* locus. (G) Allele frequencies were calculated using 1,000 bootstrap replicates for a subset of individuals grouped into five time periods (*N* = 57) (Table S9), following calculations underlying the second PBS scan. (H) Allele frequencies were calculated, five individuals were randomly resampled 1,000 times per group, for all samples within each group representing the same five time periods (*N* = 170). Groups: THRB-1: 1965–1969; THRB-2: 1970–1979; THRB-3: 1980–1989; THRB-4: 2000–2009; THRB-5: 2010–2020.

We calculated population branch statistics (PBS)^38^ using Tibetan horses as an unrelated Asian breed serving as outgroup (Figure 1A), to identify outlier genomic windows of 50 kb. Outlier regions were defined to contain at least six consecutive windows above the 99.5% quantile of the PBS distribution, providing a list of candidate loci with changing selection regime between the two Thoroughbred groups (Figure 3A and Table S10). This analysis revealed 47 candidate loci, several of which overlap with those reported in previous analyses of Thoroughbreds, such as *CAB39L*,^6^^,^^39^^,^^40^^,^^41^ *ZWINT*,^6^^,^^39^^,^^41^^,^^42^ *IL13*,^6^^,^^39^^,^^40^^,^^41^ *IL5*,^6^^,^^39^^,^^40^^,^^41^ *CYSLTR2*,^6^^,^^39^^,^^40^^,^^41^ *TRIM13*,^6^^,^^39^^,^^41^ *RCBTB1*,^6^^,^^39^^,^^40^^,^^41^ *RAD50*,^6^^,^^39^^,^^40^^,^^41^ *ARL11*,^6^^,^^39^^,^^40^^,^^41^ *IRF1*,^6^^,^^39^^,^^40^^,^^41^ and *FNDC3A*^6^^,^^39^^,^^40^^,^^41^ (Table 1). Our list of selection candidates also includes loci with reported association with traits such as athletic performance, energy metabolism, and skeletal muscle biology in non-Thoroughbred horses and other species (Table S11). Interestingly, the *MSTN* locus (otherwise nicknamed the “*speed gene*”) was not found among our selection candidates (Figure 3D), suggesting either limited statistical power, or at best modest frequency shifts during this time frame for those variants responsible for increased muscular strength and improved racing speed at short distance.^4^^,^^44^ To further explore selection signals, we calculated integrated haplotype scores (iHSs)^45^ across genomic windows of 50 kb, excluding the two genomes from the early 20^th^ century, and identified 163 selection candidates showing iHS values above the 99% quantile. This analysis returned many selection candidates also detected using PBS, such as *ZWINT*,^6^^,^^39^^,^^41^^,^^42^ *DNAJB14*,^41^ *GLIS3*,^41^ *LAMTOR3*,^41^ and *PPP1R9A*,^43^ which were previously reported as potential selection candidates in Thoroughbreds. Interestingly, many of the selection candidates corresponded again to genes associated with racing performance, such as *OCA2*,^39^^,^^41^ *BBS5*,^39^^,^^40^^,^^41^ *EBPL*,^6^^,^^39^^,^^40^^,^^41^ *GABRG3*,^39^^,^^41^ *HCN4*,^39^^,^^41^ *THSD4*,^6^^,^^39^^,^^41^ *ZPBP*,^6^^,^^41^ and *B3GALT1*^6^^,^^39^^,^^41^(Figure S11 and Table S12).

**Table 1:** Previously reported positive selection candidates in Thoroughbreds

Chromosome	Window_start	Window_end	Gene ID	Gene name
1	122520000	122570000	ENSECAG00000016415	*MYO9A*^6^^,^^39^^,^^41^
1	46580000	46630000	ENSECAG00000000633	*ZWINT*^6^^,^^39^^,^^41^^,^^42^
1	42650000	42700000	ENSECAG00000004701	*CSTF2T*^6^
4	37070000	37120000	ENSECAG00000001570	*CALCR*^43^
4	38680000	38730000	ENSECAG00000000344	*PON1*^43^
4	38570000	38620000	ENSECAG00000015961	*PPP1R9A*^43^
6	22470000	22520000	ENSECAG00000009419	*IQCA1*^6^
10	71600000	71650000	ENSECAG00000043793	*TRDN*^41^
14	42220000	42270000	ENSECAG00000008299	*IL4*^6^^,^^39^^,^^40^^,^^41^
14	42220000	42270000	ENSECAG00000009732	*IL13*^6^^,^^39^^,^^40^^,^^41^
14	42290000	42340000	ENSECAG00000010922	*RAD50*^6^^,^^39^^,^^40^^,^^41^
14	42290000	42340000	ENSECAG00000016648	*IL5*^6^^,^^39^^,^^40^^,^^41^
14	42320000	42370000	ENSECAG00000017794	*IRF1*^6^^,^^39^^,^^40^^,^^41^
14	46790000	46840000	ENSECAG00000007492	*C14H5orf63*^6^^,^^40^
14	47160000	47210000	ENSECAG00000010547	*ALDH7A1*^6^^,^^40^
14	47160000	47210000	ENSECAG00000017800	*GRAMD2B*^6^^,^^40^
14	47140000	47190000	ENSECAG00000009967	*PHAX*^6^^,^^40^
17	21040000	21090000	ENSECAG00000025521	*MIR15A*^6^^,^^39^
17	21070000	21120000	ENSECAG00000047808	*TRIM13*^6^^,^^39^^,^^41^
17	21160000	21210000	ENSECAG00000011472	*SPRYD7*^6^^,^^39^^,^^41^
17	21250000	21300000	ENSECAG00000015530	*KPNA3*^6^^,^^39^^,^^41^
17	21400000	21450000	ENSECAG00000004611	*ARL11*^6^^,^^39^^,^^40^^,^^41^
17	21410000	21460000	ENSECAG00000021791	*RCBTB1*^6^^,^^39^^,^^40^^,^^41^
17	21490000	21540000	ENSECAG00000020357	*SETDB2*^6^^,^^39^^,^^40^^,^^41^
17	21490000	21540000	ENSECAG00000014972	*PHF11*^6^^,^^40^
17	21680000	21730000	ENSECAG00000000879	*CAB39L*^6^^,^^39^^,^^40^^,^^41^
17	21690000	21740000	ENSECAG00000020688	*CDADC1*^6^^,^^39^^,^^40^^,^^41^
17	21840000	21890000	ENSECAG00000023697	*FNDC3A*^6^^,^^39^^,^^40^^,^^41^
17	21810000	21860000	ENSECAG00000035160	*MLNR*^39^^,^^41^
17	22170000	22220000	ENSECAG00000004678	*CYSLTR2*^6^^,^^39^^,^^40^^,^^41^
18	49480000	49530000	ENSECAG00000006675	*UBR3*^39^^,^^41^
18	49860000	49910000	ENSECAG00000000293	*MYO3B*^39^^,^^41^
21	17280000	17330000	ENSECAG00000014116	*SLC38A9*^39^

The *N* = 32 candidates considered are associated with outlier population branch statistic (PBS) values, as measured between a group of *N* = 2 ancient and *N* = 57 Thoroughbred horses.

In a second PBS selection scan, we disregarded PHAR and DRKN, and contrasted two groups of THRB with birth dates before and after 2000 (*N* = 31 and *N* = 26, respectively; Table S9) (Figure 3B). This analysis was set out to identify selection targets that have changed in the most recent Thoroughbred breeding history. It revealed 89 candidate loci, of which several were identified in previous selection scans (Tables 2 and S13). Notably, some candidates, such as *MYO9A*, *CSTF2T*, *GRAMD2B*, and *TRDN* overlapped with those selection candidates identified in the first PBS scan. This suggests ongoing selective shifts in the modern Thoroughbred industry, with breeding goals targeting overall similar functions pathways, albeit through partially distinct biological mechanisms. Furthermore, our candidate list also contained several genes, *SGCZ*^43^, *CNTN3*,^46^ *GRM8*,^47^ *GRIK2*,^47^ *PPP4R2*,^47^ *PDZNRN3*,^47^ and *RYBP*^48^ not identified in previous selection scans, but associated with racing and athletic performance (Table 2).

**Table 2:** Previously reported positive selection candidates in Thoroughbreds

Chromosome	Window_start	Window_end	Gene ID	Gene name
1	122450000	122500000	ENSECAG00000016415	*MYO9A*^6^^,^^39^^,^^41^
1	113580000	113630000	ENSECAG00000018053	*GABRG3*^39^^,^^41^
1	113310000	113360000	ENSECAG00000005559	*GABRA5*^39^
1	113080000	113130000	ENSECAG00000022463	*GABRB3*^39^^,^^41^
1	114660000	114710000	ENSECAG00000017677	*HERC2*^39^^,^^41^
1	114430000	114480000	ENSECAG00000009637	*OCA2*^39^^,^^41^
1	42650000	42700000	ENSECAG00000004701	*CSTF2T*^6^
3	87130000	87180000	ENSECAG00000022751	*GRXCR1*^41^
3	87430000	87480000	ENSECAG00000022873	*ATP8A1*^41^
4	82180000	82230000	ENSECAG00000019015	*GRM8*^41^^,^^42^
4	72260000	72310000	ENSECAG00000023867	*FOXP2*^41^
6	31810000	31860000	ENSECAG00000010693	*ITFG2*^41^
6	31810000	31860000	ENSECAG00000019129	*FOXM1*^41^
10	71600000	71650000	ENSECAG00000043793	*TRDN*^41^
10	53840000	53890000	ENSECAG00000020215	*GRIK2*^41^
14	47250000	47300000	ENSECAG00000017800	*GRAMD2B*^6^^,^^39^^,^^40^
16	18860000	18910000	ENSECAG00000000689	*PPP4R2*^41^^,^^46^
16	18880000	18930000	ENSECAG00000008483	*GXYLT2*^41^^,^^46^
16	18550000	18600000	ENSECAG00000014864	*PDZRN3*^41^^,^^46^
16	17750000	17800000	ENSECAG00000013575	*CNTN3*^41^^,^^46^
16	19400000	19450000	ENSECAG00000000266	*RYBP*^41^
27	17470000	17520000	ENSECAG00000000120	*SGCZ*^41^
27	33630000	33680000	ENSECAG00000051382	*DEFA31L*^41^
27	33650000	33700000	ENSECAG00000054883	*DEFA12*^41^

The *N* = 23 candidates considered are associated with outlier population branch statistic (PBS) values, as measured between the group of *N* = 31 and *N* = 26 Thoroughbred horses born before and after year 2000.

Importantly, the *MSTN* locus was again absent from the list of selection candidates (Figure 3F), aligning with the steady allele frequencies estimated among subset Thoroughbreds for various derived *MSTN* variants associated with short-distance performance, including: rs397152648 (chr18:66,608,679), rs69125012 (chr18:65,924,323), and rs69125077 (chr18:65,983,696) (Figures 3G and S12). However, these frequencies significantly increased over time when considering all individuals born between 1965 and 2020, including those related (Welch *t* test, *p* value = 10^−20^; Figure 3H). This finding is consistent with the small size genetic improvements of ∼0.05% per year measured since 1997 using average short-distance racing speeds reported for Great Britain.^49^ It also supports the model proposed by Bower and colleagues, which suggested that the allele frequency rise at *MSTN* was largely driven by the disproportionate genetic contribution of highly influential stallions such as Neartic (born 1954 in Canada) and his son Northern Dancer (born 1961), who became one of the most influential sires in Thoroughbred history and shaped modern racing bloodlines worldwide.^4^

In conclusion, by adding the genome sequence of Phar Lap, a legendary figure in Thoroughbred history, to an extensive, high-resolution genomic time-series for Thoroughbreds, we exemplify the transformative potential of historical DNA in helping unravel the complex genetic history of iconic breeds, following previous work on horses,^11^ and dogs.^50^^,^^51^ Our analyses underscore that the maintenance of a closed studbook, the further reduction of an already limited breeding stock, and mating among close relatives, were mainly responsible for the loss of genetic diversity measured. Beyond this genetic erosion, we also reveal past and ongoing selection targets that have shaped, and keep shaping, the genome of Thoroughbreds. Combined, our analyses demonstrate veterinarian archives and natural history museums as largely untapped repositories of dated genomes that can help bridge critical gaps in our understanding of the genetic history underlying breed formation and shaping the emergence of performance or production traits.

The loss of genetic diversity, historical bottlenecks, and the ongoing selection pressures documented in this study call for evidence-based breeding strategies to preserve the long-term viability of Thoroughbreds.^8^ As the industry continues to balance the pursuit of athletic excellence with genetic sustainability and animal welfare, our findings emphasize the need for genomic monitoring and diversity-aware management to mitigate further erosion of the breed genetic foundation. Future research should focus on extending the genome time-series in the early 20^th^ century and before, to assess the deeper genetic history of Thoroughbreds during the 19^th^ century and back to the bloodline studbook creation in the late 18^th^ century when breeding preferences favored athletic performance in older horses and over longer distances. Future work shall also prioritize functional characterization of the putative selection candidates identified here, which may provide further insights into breeding decisions and selection criteria within the modern Thoroughbred racing industry.

### Limitations of the study

While this study leverages a large, time-stamped genome series, critical temporal gaps persist. This is especially true for the first half of the 20th century, which is represented by only two Thoroughbred genomes. This sparsity constrains the statistical power of genome-wide scans aimed at detecting shifting selection pressures over the past two centuries. Additionally, the deeper historical origins of the Thoroughbred bloodline remain unsampled at the genomic level, limiting insights into the breed foundation and early evolution. Furthermore, Thoroughbred breeding is highly regionalized, with distinct populations in the United States, Australia, Britain, and Asia. Future research should evaluate whether the temporal trends observed here extend across all regions, and address potential biases introduced by population structure in our selection scans. It should also aim at functionally validating the multiple selection candidates identified.

## Resource availability

### Lead contact

Further information and requests for resources and reagents should be directed to and will be fulfilled by the lead contact, Ludovic Orlando (ludovic.orlando@utoulouse.fr).

### Materials availability

This study did not generate new, unique reagents.

### Data and code availability

All raw sequencing data produced in this study have been deposited at the European Nucleotide Archive (ENA, Accession Nb. PRJEB111649). This study does not report original code. Any additional information required to reanalyze the data reported in this study is available from the lead contact upon request.

## Acknowledgments

This work was supported by the 10.13039/100018693European Union’s Horizon Europe program under the Marie Skłodowska-Curie Actions Postdoctoral Fellowship (PostEquus, HORIZON-MSCA-2023-PF-01, grant no. 101146226, type of action: HORIZON-TMA-MSCA-PF-EF). L.O. has received funding from the 10.13039/501100000781European Research Council (10.13039/100017325ERC) under the 10.13039/100018693Horizon Europe (grant agreement no. 101071707-Horsepower) research and innovation program.

## Author contributions

Conceptualization, J.N.M., K.N., T.K., and L.O.; methodology, T.K. and L.O.; investigation, H.A.N., A.S.-O., J.N.M., T.K., and L.O.; formal analysis, H.A.N., A.S.-O., and L.O.; writing – original draft, L.O., with input from H.A.N. and A.S.-O.; writing – review and editing, H.A.N., A.S.-O., J.N.M., K.N., T.K., and L.O.; resources, J.N.M., K.N., T.K., and L.O.; visualization, H.A.N., with input from L.O.; and supervision, L.O.

## Declaration of interests

The authors declare no competing interests.

## STAR★Methods

### Key resources table

**Table:** 

REAGENT or RESOURCE	SOURCE	IDENTIFIER
**Biological samples**		

Phar Lap hair	This Study, provided by Tommy Woodcock	Ages_1_8005

**Chemicals, peptides, and recombinant proteins**		

Proteinase K	Sigma-Aldrich	Cat#3115844001
Accuprime^TM^ Pfx DNA polymerase	Invitrogen	Cat#12344-024

**Critical commercial assays**		

MinElute PCR Purification Kit	QIAGEN	Cat#28006
Uracil-Specific Excision Reagent	New England Biolabs Inc.	Cat#M5505
Amicon Ultra-4 30 kD	Millipore	Cat#UFC803024
AMpure XP Beads	Agencourt	Cat#A63882
Qubit dsDNA HS Assay	Invitrogen	Cat#Q32854
High Sensitivity D1000 ScreenTape Assay	Agilent	Cat#5067-5584

**Deposited data**		

ENA	This study	PRJEB111649

**Other**		

TapeStation 2200	Agilent	Cat#G2964AA
QuBit 4	Invitrogen	Cat#Q33238

### Experimental model and study participant details

No experimental procedures involving living animals were conducted. The biological material analyzed consisted of six tail hair samples from Phar Lap, the renowned Australian Thoroughbred racehorse (1926–1932). The hair samples were preserved in a sealed envelope authenticated by Tommy Woodcock, Phar Lap’s strapper and primary caretaker, and have remained in the family of one of the co-authors (K.N.) since their collection. DNA was extracted from these historical specimens in dedicated ancient DNA laboratories following strict contamination-control procedures.

### Method details

#### General comment

DNA extraction, library building, indexing and PCR amplification was conducted in the state-of-the-art facilities of the Center for Anthropobiology and Genomics of Toulouse (CAGT), University of Toulouse, France. Pre-PCR amplification steps were performed in wet-lab facilities strictly dedicated to ancient samples processing. These facilities are physically located in an independent building, separated by a 2-min walk from laboratories where post-PCR amplification and modern DNA samples are manipulated. Standard measures aimed at limiting any risk of DNA contamination were strictly followed, including: decontamination of all surfaces and equipment using bleach or DNA AWAY before and after each procedure; use of disposable personal protective equipment; single-use DNA-free plastic ware; inclusion of negative controls (blank reactions) during DNA extraction, library building and PCR enrichment steps. Of note, to prevent cross-contamination, no other sample was processed in the same session as the PHAR hair samples, until the underlying libraries were pooled for deep-sequencing with other triple-indexed sequencing libraries. Crucially, except the DRKN samples, which was processed several years ago by another experimenter,^17^ no Thoroughbred specimens were extracted for DNA in the CAGT facilities, ruling out other possible contamination sources.

#### Ancient DNA extraction

PHAR samples consisted in six tail hair fragments (∼10–15 cm each), which were given to Cliff and Thelma Hinchliffe in 1983 by Tommy Woodcock. In 2014, the samples were passed from her father to one of the co-authors (KN). The official statement documenting the history of ownership was drawn up and signed by KN, her father, and a witness on August 16, 2019.

DNA extraction was performed following a protocol modified from Rasmussen and colleagues^52^ and described in full detail by Taylor and colleagues.^53^ Briefly, hair samples were decontaminated in a fresh 0.5% sodium hypochlorite solution and rinsed three times in molecular grade water, before incubation for 18 h at 42°C in 4 mL digestion buffer (10 mM Tris, 10 mM NaCl, 5 mM CaCl_2_, 2.5 mM EDTA, 1% SDS, 100 mM DTT and 2 mg/mL proteinase K). After 2 min of centrifugation at maximum speed, the supernatant was transferred on an Amicon Ultra-4 filter (Millipore) and centrifuged at 3000 rpm until its volume was reduced to 250 μL. The concentrated supernatant was collected and purified on a single MinElute column (QIAGEN), following manufacturer’s instructions and eluted in 48 μL pre-heated elution buffer (QIAGEN EB, 0.05% Tween).

#### Removal of uracil-residues

The DNA extract obtained was divided in two aliquots and subjected to Uracil-Specific Excision Reagent (USER) treatment (22.3 μL DNA extract and 7 μL USER enzymatic mix, incubation for 3h at 37 C), following the methodology from Fages and colleagues.^17^

#### Sequencing library building and amplification

Four main independent DNA libraries were prepared, following a method described in Fages and colleagues^17^ and Lira Garrido and colleagues.^54^ The procedure relied on the ligation of indexed blunt-end adapters on double-stranded ancient DNA inserts (14.9 μL of USER-treated extract as input), and was slightly adapted from the protocol originally developed by Meyer and Kircher.^55^ Two 7-nucleotide-long index sequences were selected from the list provided by Rohland and colleagues^56^ to tag both P5 and P7 adapters. Each DNA library was subjected to three independent PCR amplifications for 11–13 cycles by the Accuprime Pfx DNA polymerase (Thermo Fisher Scientific), using 3 or 4 μL of unamplified library (in a total reaction volume of 25 μL or 50 μL, respectively) and indexed PCR primers, as reported in Fages and colleagues^17^ and Lira Garrido and colleagues.^54^ This provided a total of 12 independent library amplifications for sequencing. After Ampure© beads purification, amplified and triple-indexed DNA libraries were quantified and assessed for their fragment size distribution using a QuBit fluorometer (Invitrogen, high sensitivity dsDNA HS assay) and a TapeStation (Agilent, High Sensitivity D1000 screen tape), respectively.

#### Illumina sequencing

DNA libraries were pooled with other triple-indexed libraries on three different pools, and sequenced on the Illumina NovaSeq 6000 instruments from Novogene Europe (paired-end mode, 2 × 150). No sequencing index was used more than once in a given pool.

#### Comparative genome panel

Publicly available FASTQ files for two ancient samples DRKN and ALFT were downloaded from the European Nucleotide Archive (ENA, Accession Nb. PRJEB31613 and PRJEB71445, respectively).^17^^,^^22^ Previously published genome sequences from global modern horses (*N* = 850) were obtained from both ENA and the Sequence Read Archive (www.ncbi.nlm.nih.gov). Information regarding the samples analyzed in this study can be found in Table S3.

#### Alignment, and trimming of sequencing reads

Read pairs for both modern and ancient individuals were trimmed and aligned to the horse reference genome (EquCab3.0),^57^ following the procedures described by Librado and colleagues.^22^^,^^28^ For each individual, AdapterRemoval2 (version 2.3.0)^58^ was used to demultiplex DNA libraries, trim Illumina adapter sequences and collapse paired-end reads showing sufficient sequence overlap, while further trimming those with low-quality ends and removing those resulting templates shorter than 25 nucleotides (--collapse --minlength 25 --trimns --trimqualities --minadapteroverlap 3 --mm 5). For demultiplexing, the list of expected indexes was provided using the --barcode-list flag, tolerating at most one mismatch per index (--barcode-mm-r1 1 --barcode-mm-r2 1). Collapsed and uncollapsed read pairs were subsequently processed using the Paleomix bam_pipeline^59^ (v1.2.13) for alignment with Bowtie2^60^ (v2.3.4.1) against the EquCab3.0 reference genome. Mapping parameters followed the recommendations from Poullet and Orlando,^61^ with local realignment around indels carried out using GATK’s IndelRealigner.^62^ Finally, sequencing reads representing mapping quality score below 25, and/or showing PCR duplicates were disregarded.

For ancient DNA data generated for PHAR, mapDamage2^63^ was applied to quantify postmortem DNA damage patterns, sampling 100,000 reads randomly from each library. We then followed the procedure from Librado and colleagues^22^ for reducing the impact of postmortem DNA decay on the quality of sequence data. Briefly, using the parameters “threshold 1; DAM” and “upper threshold 1; NODAM” in PMDtools^64^ (v0.50), reads likely to contain postmortem DNA (PMD) damage were excluded (DAM-aligned) and stored separately from undamaged (and NODAM-aligned) reads. DAM reads underwent rescaling with mapDamage (penalizing all transitions) and then trimmed by 10 bp at both ends, whereas, NODAM reads were trimmed for 5bp at their ends and combined with DAM reads.

#### Variant discovery, phasing and filtering for modern horse variants

Using the methodology of Todd and colleagues,^11^ variant calling for SNPs and deletions or insertions (INDELs) were carried out on the subset of *N* = 850 high-coverage modern genomes. As part of this procedure, Graphtyper^23^ (v2.7.6) was run for each chromosome to call variants, retaining only those SNPs passing the quality thresholds proposed by Eggertsson and colleagues,^23^ i.e., ABHet <0.0 | ABHet >0.33, ABHom <0.0 | ABHom >0.97, MaxAASR >0.4, and MQ > 30. The “vcffilter” function from Vcflib^65^ was used to filter low-quality variants. Those resulting SNPs were then further filtered using a combination of GATK and BCFtools^66^ (v1.21) to retain only biallelic variants (alleles = 2) and to meet the following thresholds: minor allele frequency, MAF≥0.01; Hardy-Weinberg equilibrium P-value≥0.001; Phred-score≥20, and; genotype missingness≤0.2. Unassembled contigs, the X chromosome, and INDELs were removed, leaving a total of *N* = 16,655,519 SNPs along the 31 autosomes for further analysis. Those genotype variants were phased with *BEAGLE*^67^ (v5.0) using the recombination map from Beeson and colleagues.^68^

### Quantification and statistical analysis

#### Genotype imputation

To generate high-quality genotype calls from the relatively limited sequencing data available for the three ancient individuals (PHAR, DRKN, and ALFT), we used GLIMPSE2,^25^ which employs a Gibbs sampling approach that iteratively alternates between phasing and haploid imputation steps, on the sequence data underlying the three ancient samples. First, we divided autosomes into chunks using the sequential algorithm from *GLIMPSE_chunk* (--sequential) with default parameters. Next, imputation was carried out on the generated chunks using *GLIMPSE_phase* with the parameters --mapq 25 and --baseq 30, and turning on the options --keep-orphan-reads --ignore-orientation. For this step, the phased complete dataset of *N* = 16,621,426 high-quality SNPs for *N* = 850 modern horse genome as the reference panel. Finally, the imputed chunks were merged, first per autosome, then across the whole genome, using *GLIMPSE_ligate* with default parameters.

#### Imputation accuracy evaluation

Imputation accuracy and genotype concordance was assessed using *GLIMPSE_concordance* and a shortlist of *N* = 10 modern Thoroughbred genomes (Table S4) that were downscaled to the average coverage-of-depths measured in the three ancient individuals. These 10 samples showed the highest average coverage-of-depths and provided a representation of the diversity within Thoroughbreds. Downscaling was carried out applying Samtools^66^ (v1.3.1) on the corresponding genome BAM files. The full, original data underlying those individuals were excluded from the reference matrix of phased genotypes before imputation was performed on their downscaled sequence data, following the same procedure as above. For those individuals, correct genotypes were considered to be those genomic sites supported by at least eight reads and genotype posterior probabilities ≥0.99 in the original high-quality reference panel of phased genotypes. Several metrics were calculated for assessing the overall imputation accuracy, including genotype mismatch rates (%, for RR, RA, and AA genotypes, where R and A mean reference and alternate allele, respectively), imputation uncertainty (R^2^ error), non-reference discord (%), as well as genotype and dosage R^2^ errors, as originally defined by Rubinacci and colleagues^25^ (Figure S2). Allele frequency bins for the computation of *r*^2^ (imputation accuracy) were set at 0.00, 0.01, 0.025, 0.05, 0.075, 0.1, 0.25, and 0.5 (Figure S2).

#### Population structure

A Neighbor-Joining phylogenetic tree was generated based on pairwise genetic distances among *N* = 853 individuals (*N* = 26,051,764 SNPs). Distances were first computed with PLINK^69^ (v1.9) using the --distance square 1-ibs flat-missing option, before inferring the tree topology using the bioNJ algorithm implemented in FastME^70^ (v2.0). Support values for nodes were obtained from 100 bootstrap pseudo-replicates (sampling an equivalent number of SNPs with replacement), employing the topology refinement parameter (-n) (Figure S4).

Individual ancestry proportions were inferred with ADMIXTURE^26^ (v1.3.0), using cross-validation (CV) error to identify the optimal number of genetic clusters (*K*). SNPs (*N* = 26,051,764) were first pruned for linkage disequilibrium in PLINK (--indep-pairwise 50 10 0.2), leaving *N* = 2,642,046 variants. ADMIXTURE was run for *K* values between 2 and 10 on *N* = 853 samples (excluding outgroup), and the CV error indicated that *K* = 9 (0.3072) provided the best fit to the data (Figure S5). In order to visualize genetic affinities, PCA was conducted using SmartPCA,^71^ implemented in the EIGENSOFT package on the subset of *N* = 2,642,046 pruned variants (Figure S3). To assess the amount of shared genetic drift between modern and the three ancient samples PHAR, DRKN and ALFT on the one hand, and any modern breed or population, we performed *f*3-outgroup statistics using Przewalski’s horses (*N* = 23) as the outgroup, following the form *f*3 (ancient, modern; outgroup), with ADMIXTOOLS^72^ (v5.0).

ROHs were called for each individual in our dataset individually using the –homozyg function in PLINK v1.9^69^ and the following parameters: --geno 0.01, --homozyg-window-het 1 and --homozyg-window-missing 5, --homozyg-window-snp 50, --homozyg-density 50, --homozyg-gap 1000, --homozyg-window-threshold 0.05, --homozyg-snp 100, --homozyg-kb 100. We then divided stratified ROHs into four groups based on their length: short (≥100 kb and <500 kb), intermediate (≥500 kb and <1 Mb), long (≥1 Mb and <2 Mb) and very long (≥2 Mb) and calculated the total genomic length encompassing these three groups in each individual genome. In addition, extremely long ROH (>5 Mb) were identified in each individual to enable comparison with previous studies. Individual heterozygosity rates were calculated using the “--het” option from PLINK v1.9. Finally, to complement heterozygosity estimates, we also calculated nucleotide diversity (π) for breeds represented by at least five individuals.

#### Demographic inference

We first used KING^73^ to infer relationships (--kinship) in the Thoroughbred breed sample (*N* = 328) and identified *N* = 271 individuals that were related up to the 6^th^-degree. These individuals were excluded resulting in a final dataset of 57 individuals. We then used GONE^29^ to estimate the trend of recent effective population size over time. We performed 100 independent replicates, in which 50,000 SNPs were randomly selected from each chromosome for every analysis. Analyses were conducted using all Thoroughbred horses combined, as well as separately for selected subsets of individuals born before 2000 (*N* = 31) and those born in or after 2000 (*N* = 26). We investigated Ne changes within 200 generations, a period recognized as reliable by the GONE developpers (see GONE User’s Guide (https://github.com/esrud/GONE).

#### Exploration of selective sweep regions

We used ANGSD^74^ to calculate PBS,^38^ which estimates the branch lengths of two focal populations with respect to one outgroup and has been shown to be effective for detecting recent natural selection. In a first analysis, we used PHAR and DRKN to represent ancient Thoroughbred horses, and the subset of *N* = 57 (Table S9) modern Thoroughbreds to serve as modern (≥1965) individuals. These historical genomes were included to provide temporal context for allele frequency changes during the development of modern Thoroughbreds, although we note that the limited number of early genomes precludes population-level inference for this period. Tibetan horses (*N* = 8) were designated as the outgroup due to their PCA placement (Figure S3) and phylogenetic divergence (Figure S4).

In a second analysis, we excluded the ancient horses and split the group of modern Thoroughbred into two temporal groups, i.e., those born before and after year 2000. Calculations were carried out within 50 kb sliding windows, with a step-size of 10 kb. We used the stringent 0.05% empirical threshold for detecting outlier PBS values to obtain a conservative list of selection candidates (Figure 3). Additionally, we carried out an independent within-population selection scan using the iHS to further validate candidate loci. The analysis was conducted considering the set of 57 modern Thoroughbred horses, and using sliding windows of 50 kb across the genome, and the resulting iHS values were normalized (--norm) to account for allele frequency differences and to enable genome-wide comparison of selection signals.

#### Frequency for MSTN

To investigate temporal changes in the *MSTN* locus, we estimated allele frequencies at different genomic positions (rs397152648 (chr18:66,608,679); rs69125012 (chr18:65,924,323); and rs69125077 (chr18:65,983,696)) across five generations of Thoroughbred horses (Figures 3G, 3H, and S12). Allele frequencies were first calculated using a subset of samples, consistent with the dataset employed for the PBS scan, and uncertainty was assessed using 1000 bootstrap replicates. In addition, allele frequencies were estimated using all available samples per generation; to account for unequal and small sample sizes among groups, five individuals were randomly resampled 1000 times within each generation. Together, these analyses provide a robust assessment of temporal shifts in *MSTN* allele frequencies while minimizing the effects of relatedness and sampling heterogeneity.

## Supplemental information

Document S1. Figures S1–S12

Table S1. Modern Thoroughbred samples used in this study

Table S2. Thoroughbred subgroup classification (THRB-1 to THRB-5) based on year of birth, related to Figure 1E

Table S3. Modern sample sizes by breed/subpopulation included in this study

Table S4. List of samples used to assess imputation accuracy

Table S5. Individual-level genetic diversity metrics, including observed heterozygosity (H_obs) and genomic inbreeding coefficient (F)

Table S6. Genome-wide nucleotide diversity (π) across different breeds The column labeled “*N*” reports the number of genomes in each category. Breeds represented by fewer than five genomes were excluded from the analysis.

Table S7. Significance levels of pairwise comparisons between the FROH values measured between different age groups, related to Figure S7

Table S8. Individual-level runs of homozygosity (ROH) and corresponding FROH estimates across different ROH length classes

Table S9. Modern Thoroughbred subgroups (N = 57) used for this study

Table S10. Positively selected genes identified between ancient samples and modern Thoroughbred horses, ranked within the top 0.5% based on PBS analysis, related to Figure 3A

Table S11. Selection candidates corresponding to loci previously associated with traits such as athletic performance, energy metabolism, and skeletal muscle biology in non-Thoroughbred horses or other species

Table S12. Positively selected genes identified in a subset of N = 57 modern Thoroughbred genomes, using the iHS method44

Table S13. Positively selected genes identified between modern Thoroughbred horses before and after 2000, ranked within the top 0.5% based on PBS analysis, related to Figure 3B
