# Supplementary material for: Demographic change and selection patterns in thoroughbred racing horses over the 20th century
Source: iScience. 2026 Jul 17;29(8):116830. doi: 10.1016/j.isci.2026.116830 (PMC13400855; doi:10.1016/j.isci.2026.116830)
Supplement: Document S1. Figures S1–S12 [file mmc1.pdf]

iScience, Volume *29*

Supplemental information

Demographic change and selection patterns in thoroughbred racing horses over the 20th century

Hojjat Asadollahpour Nanaei, Andaine Seguin-Orlando, Kerry Negara, James N. MacLeod, Ted Kalbfleish, and Ludovic Orlando

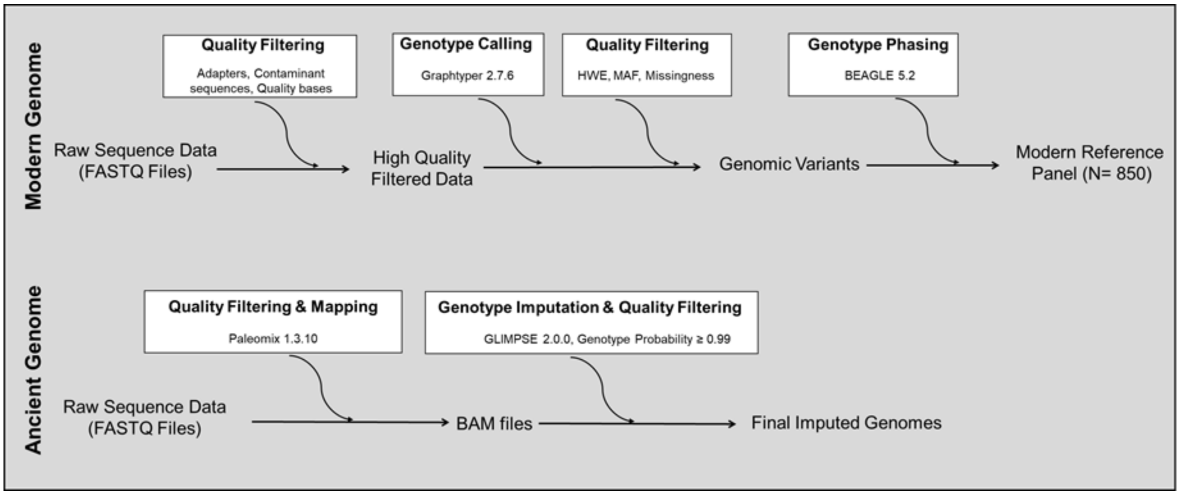

Figure S1. Workflow for modern variant discovery and phased genotype imputation in ancient horses. Flowchart illustrating the different sequential steps followed for identifying modern variants (top), and imputing phased genotypes for three ancient individuals, corresponding to Figure 1. Variant discovery and imputation were based on the biallelic SNP variation present in a phased reference panel comprising N=850 modern horses.

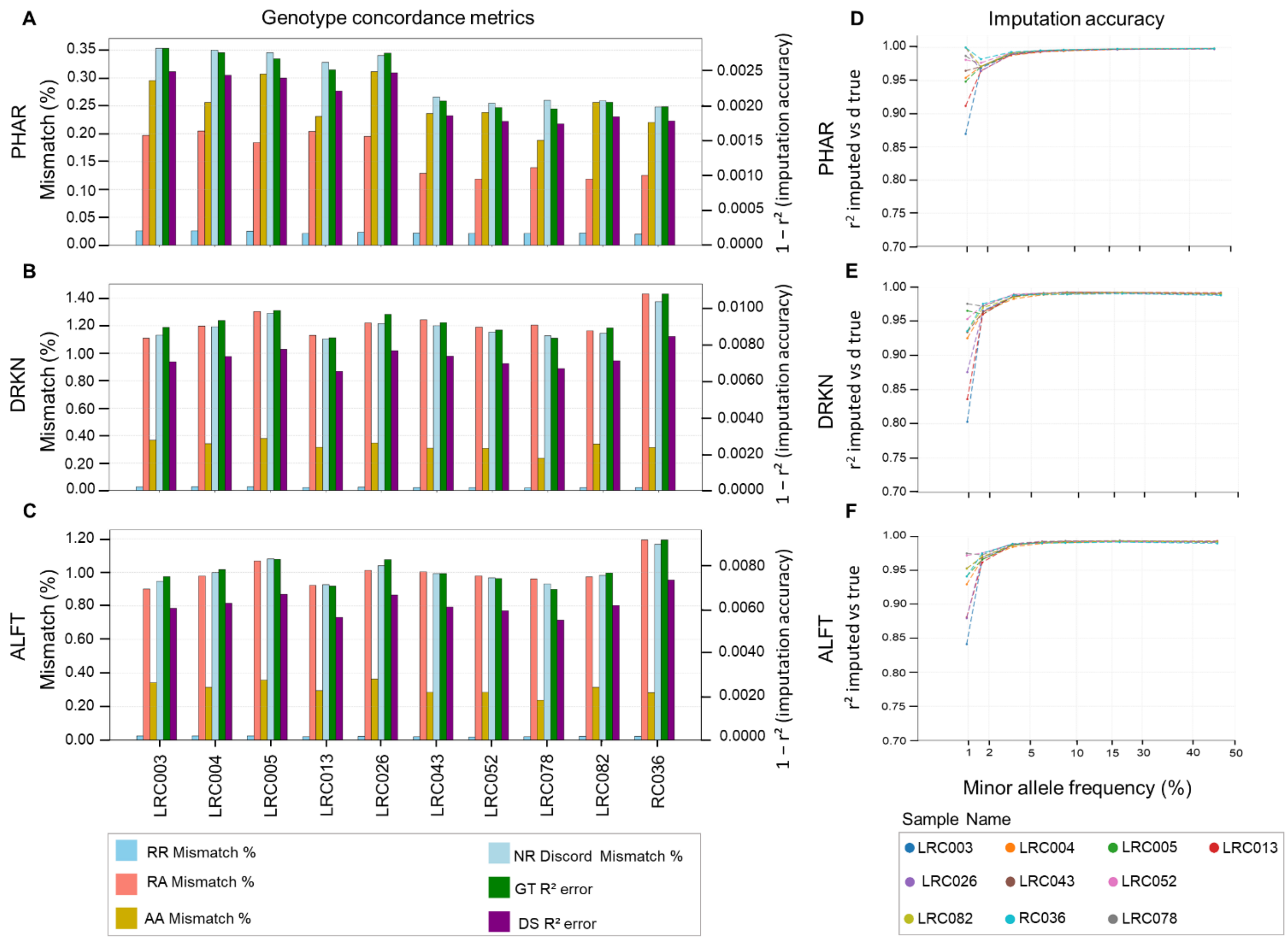

Figure S2. Imputation quality assessment for the three ancient individuals. (A-C) Genotype concordance metrics. Bars represent per-sample genotype mismatch rates (left Y-axis, %), and imputation uncertainty (right Y-axis, R^2 error=$1-R^2$). RR mismatch (%), RA mismatch (%), and AA mismatch (%) indicate genotype discordance rates for homozygous reference, heterozygous, and homozygous alternate genotypes, respectively. Non-reference (NR) discord mismatch (%) reflects the overall discordance among non-reference genotypes. GT R^2 error and DS R^2 error represent imputation accuracy loss for best-guess genotypes and dosage values, respectively, with lower values indicating higher imputation accuracy. (D-F) Imputation accuracy (r^2) as a function of minor allele frequency (MAF). The 10 down-sampled modern Thoroughbred genomes are shown on the x-axis of panels (A-C), and on the legend of panels (D-F).

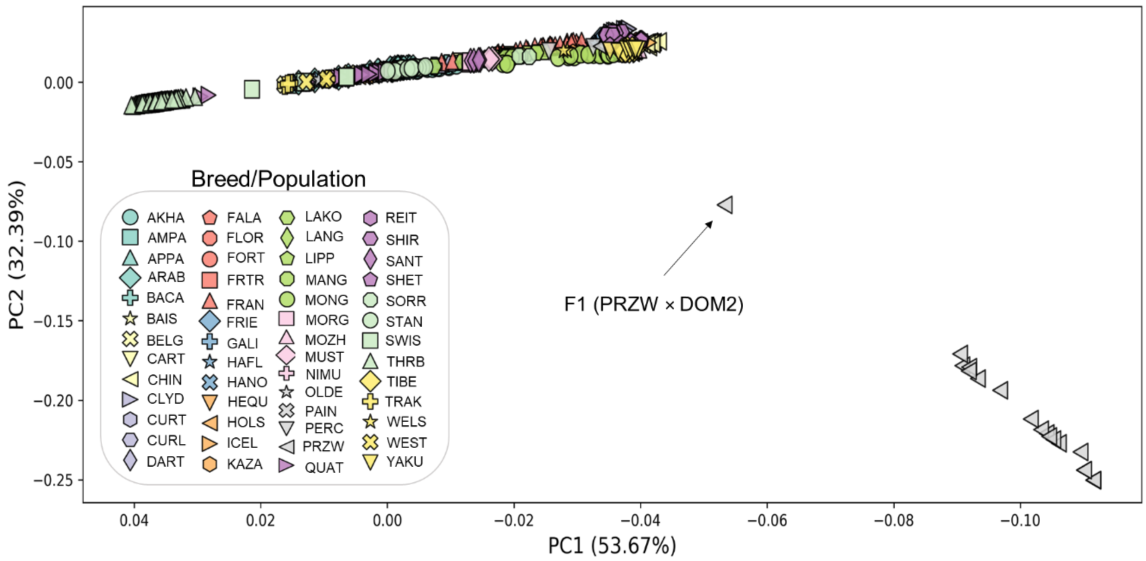

Figure S3. Principal component analysis (PCA) for the entire genome panel. The analysis comprised N=850 modern horses, including N=23 Przewalski's horses, PRZW, and one first-generation hybrid between a PRZW and a domesticated horse. PCA was carried using the "SmartPCA[71]" function implemented in the EIGENSOFT package (v6.1.4), and considering N=2,642,046 biallelic SNPs (MAF≥0.05%).

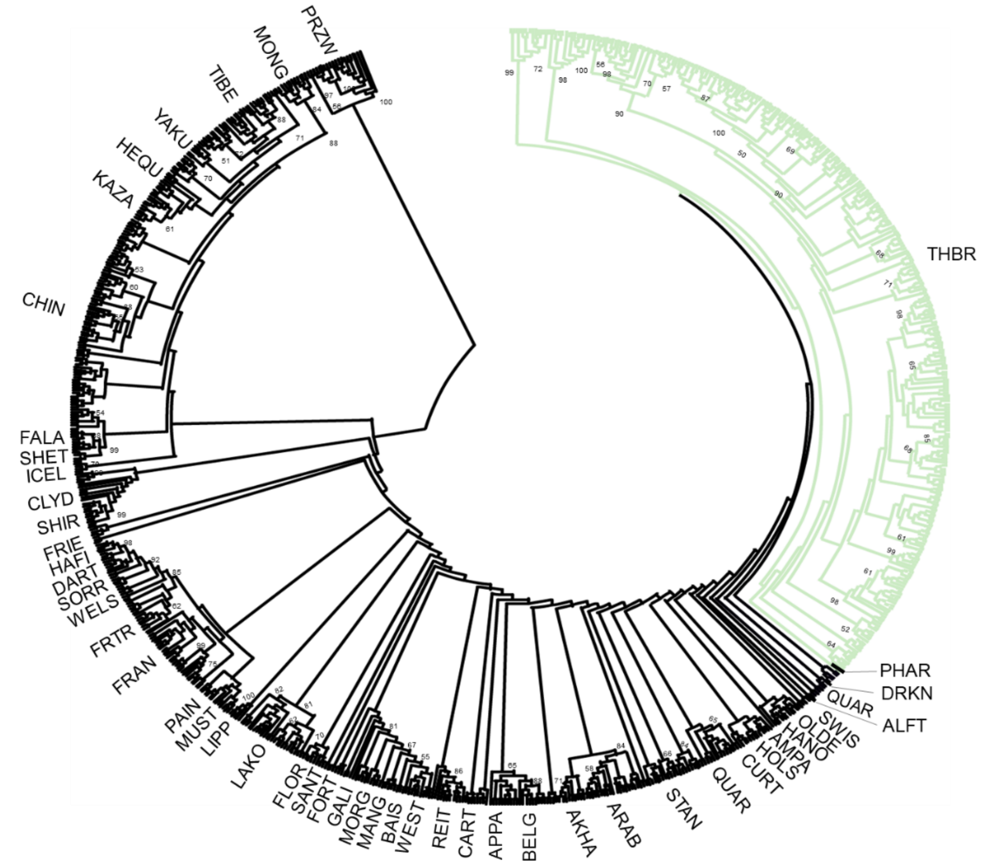

Figure S4. Neighbor-joining (NJ) phylogenetic inference. The tree was reconstructed using the BioNJ algorithm from FastME[70] version 2.1.4 and 100 bootstrap pseudo-replicates (N=853 individuals, including N=850 modern horses, and N=3 ancient individuas). Pairwise genetic distances were calculated from N= N=26,051,764 biallelic SNPs with PLINK[69] v1.9. Major breeds and populations are labeled, together with three ancient samples, according to the correspondence provided in Table S1.

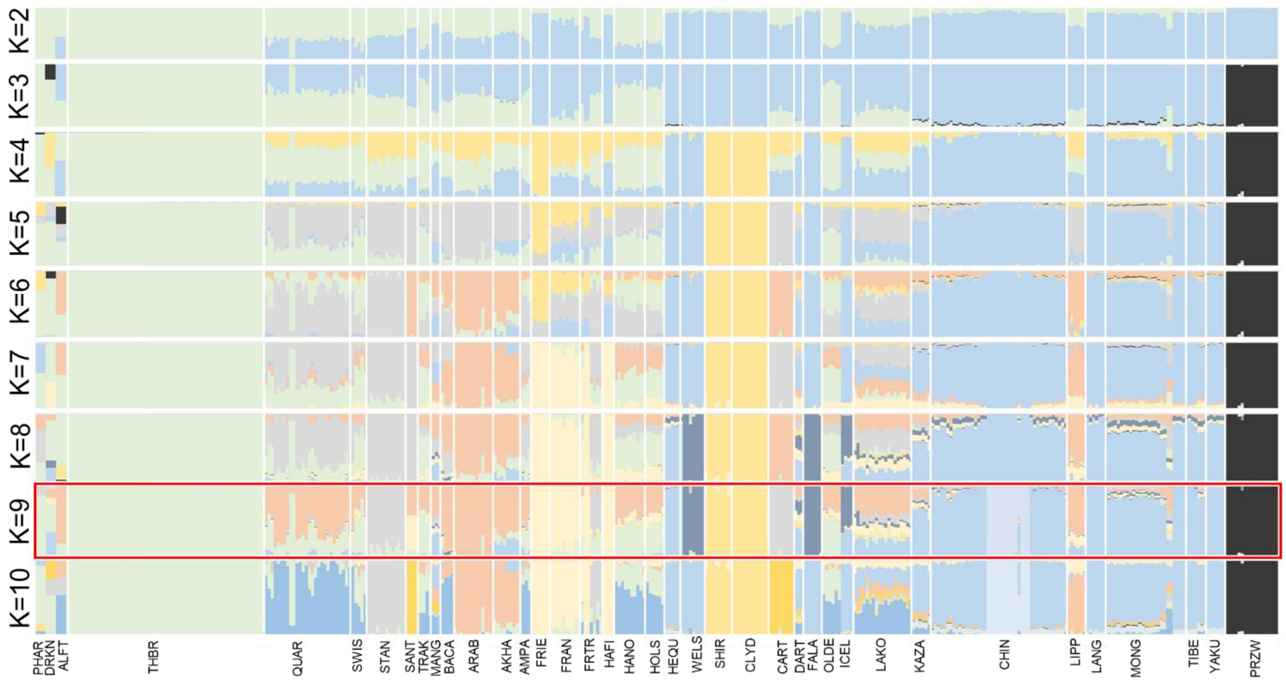

Figure S5. Admixture[26] (v1.3.0) genetic ancestry profiles. The proportions of K ancestry components for K=2 to K=10 are shown vertically, from top to bottom, related to Figure 1. The identity and number of horses in each subpopulation are outlined in Table S3. The cross-validation (CV) error for the best number of K ancestry components (K=9) was 0.30727.

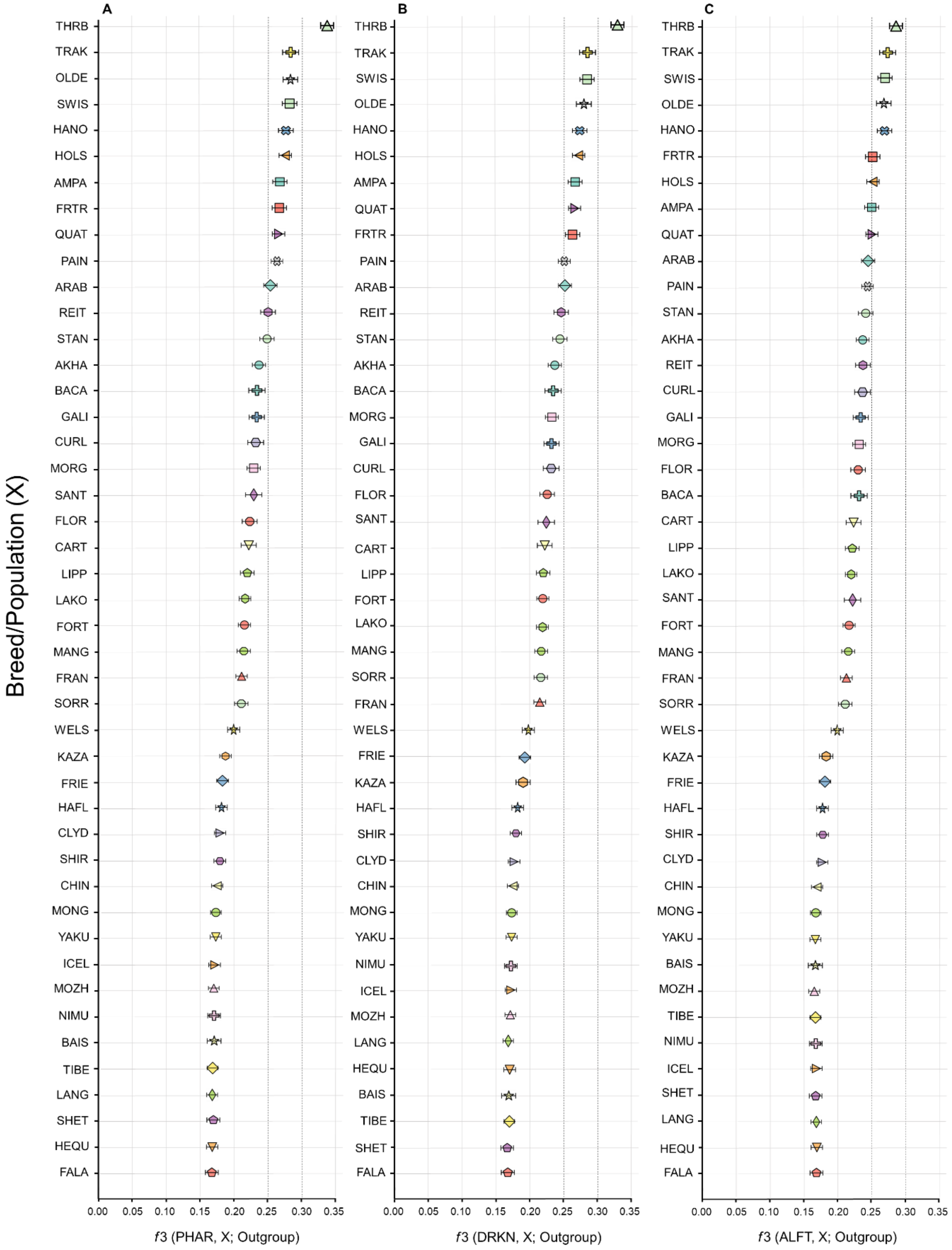

Figure S6. *f*3-outgroup statistics. The amount of shared genetic drift between three ancient samples (PHAR, DRKN and ALFT) and groups of modern horse breeds (X) are estimated using *f*3-outgroup statistics calculated with ADMIXTOOLS[72] (v 8.0.2), relative to N=23 Przewalski horses representing the outgroup. *f*3-outgroup values are sorted to represent the closest breeds or populations present in our reference panel on the top of the figure.

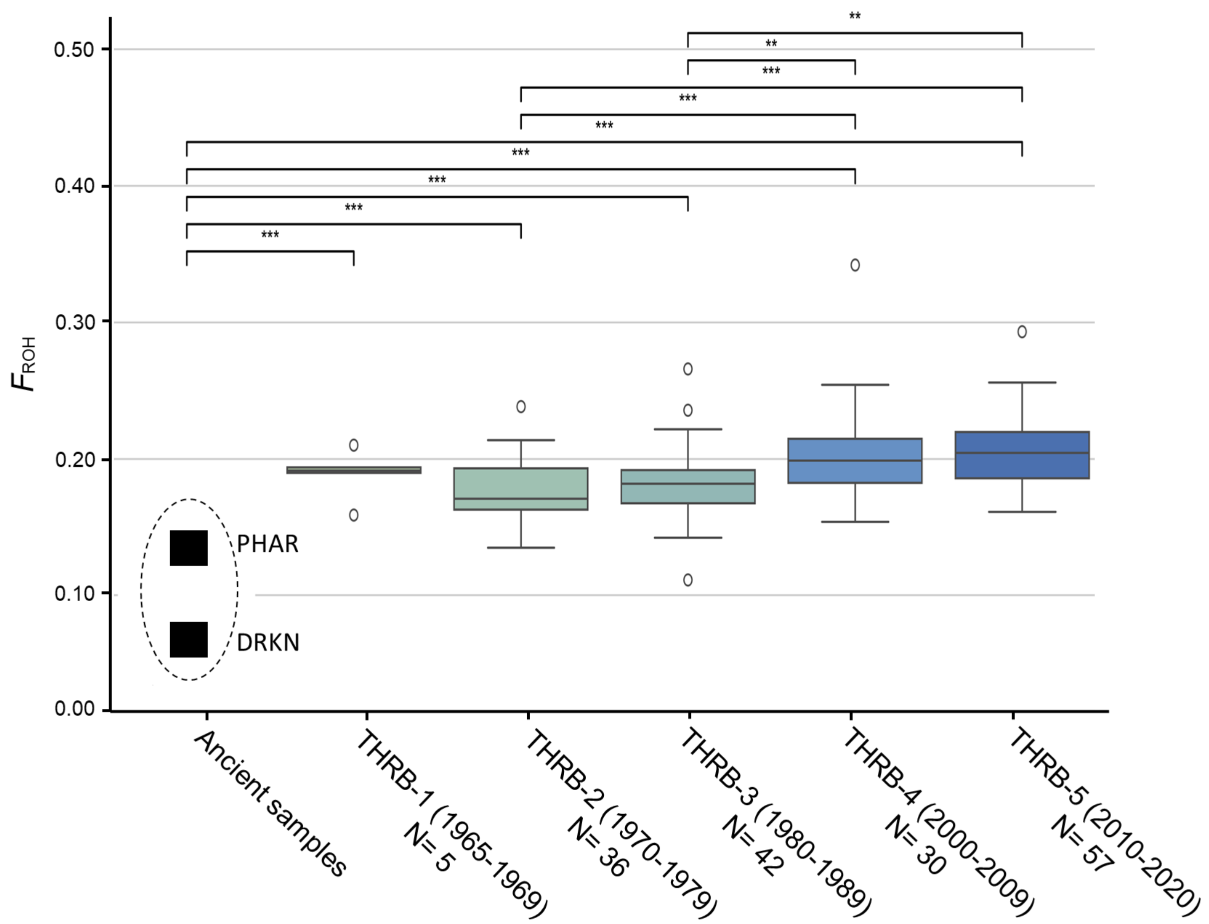

Figure S7. Boxplots showing the distribution of F_{ROH} values across different groups of Thoroughbred horses, sorted by decade. Significant pairwise differences between groups were determined using Tukey's HSD test and are indicated by stars (*** $p < 0.001$, ** $p < 0.01$, * $p < 0.05$, ns not significant). The two ancient samples (PHAR and DRKN) are colored in black. Boxplots represent the 25%, 50% and 75% quantiles, with upper and lower whiskers showing values within the 1.5 interquartile range.

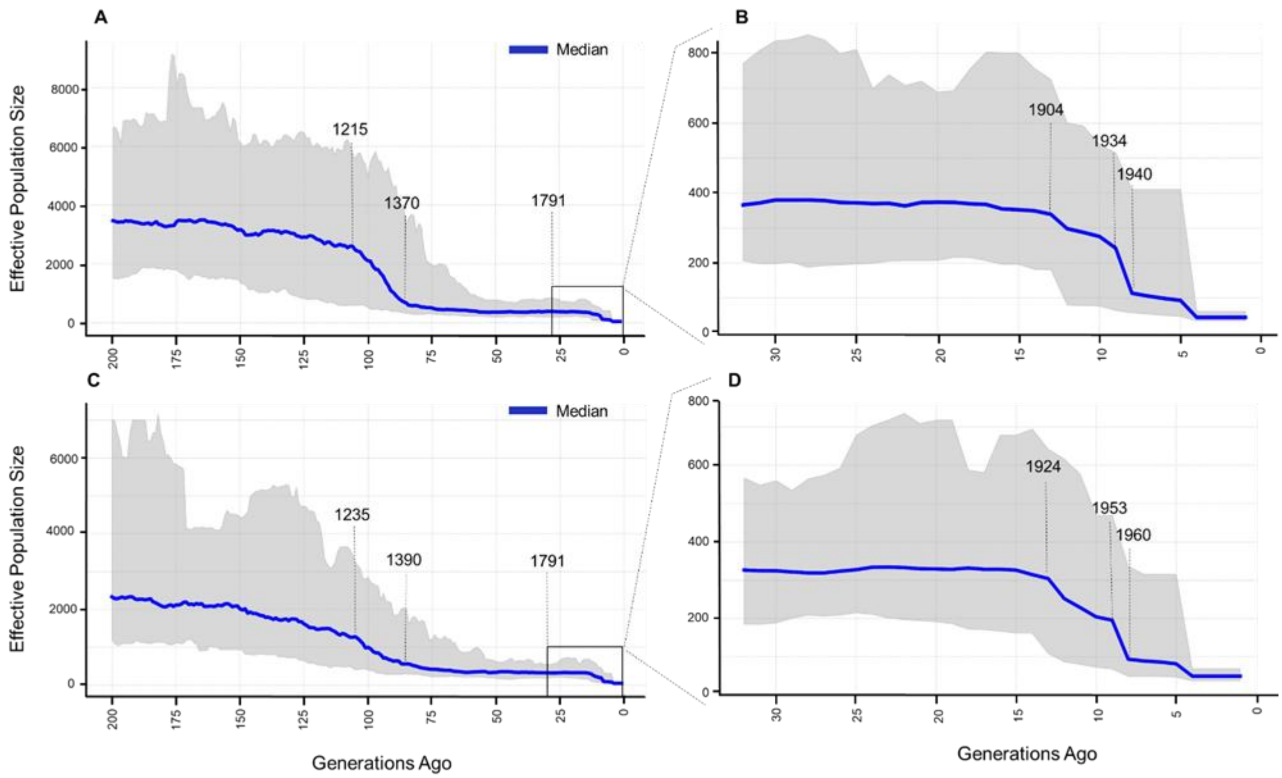

Figure S8. Demographic temporal trajectory of the Thoroughbred bloodline. The different panels show the demographic trajectory resulting from GONE[32] modeling, providing population effective sizes in the 200 generations preceding the sample set considered: individuals born before (A) or after (C) year 2000. Panels (B) and (D) provide zoomed-in inlets from panels (A) and (C), focusing on the ~30; latest generations.

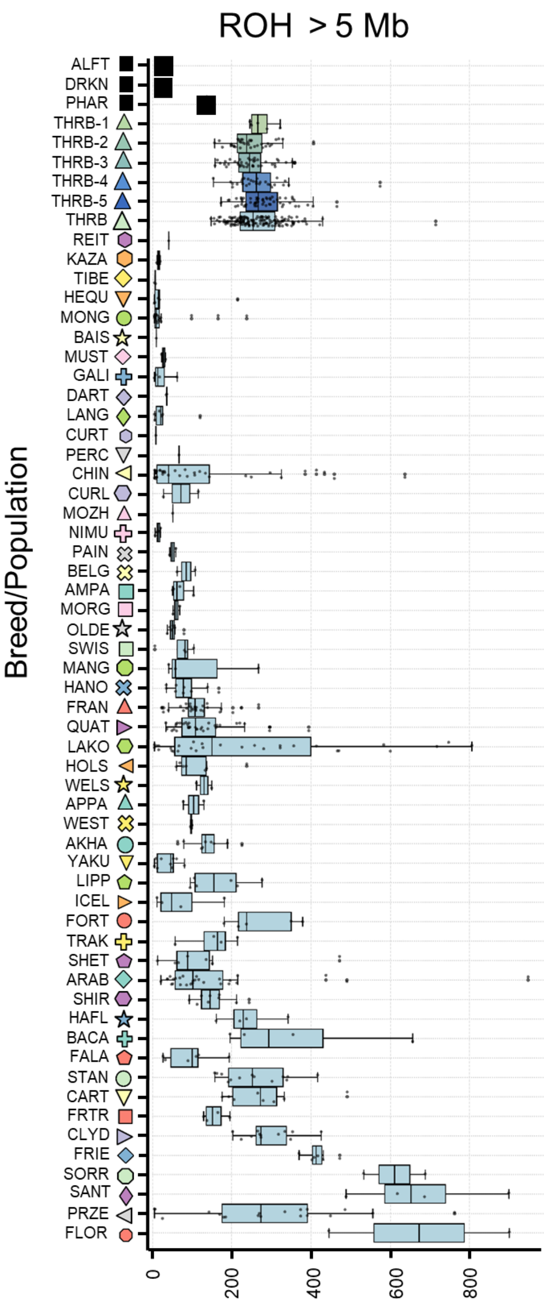

Figure S9. Total length of runs of homozygosity (ROH) ≥ 5 Mb across different populations/groups.

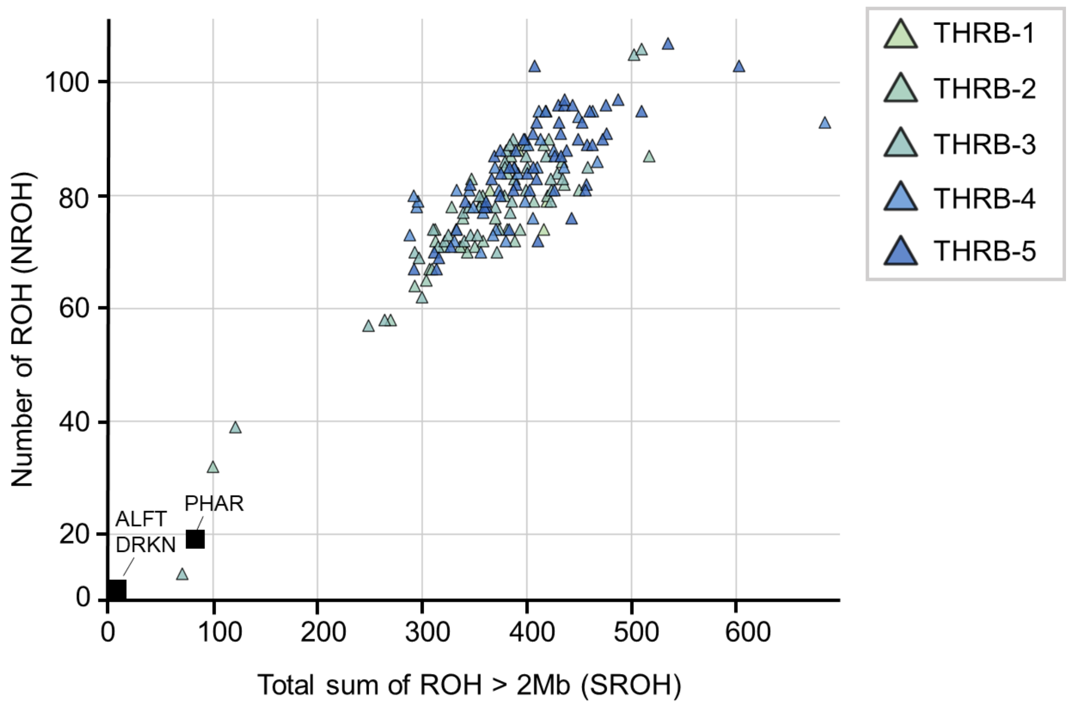

Figure S10. Number of ROH versus total sum of ROH length (>2Mb) across Thoroughbred subgroups. The plot shows relationship between the number of runs of homozygosity (ROH) and the total ROH length (>2 Mb) across Thoroughbred subgroups. Each point represents an individual genome. Colored triangles indicate modern Thoroughbred subgroups, while black squares highlight ancient samples (ALFT, DRKN, and PHAR). Both ALFT and DRKN show no detectable ROH (>2 Mb), whereas PHAR exhibits a low ROH burden compared to modern individuals.

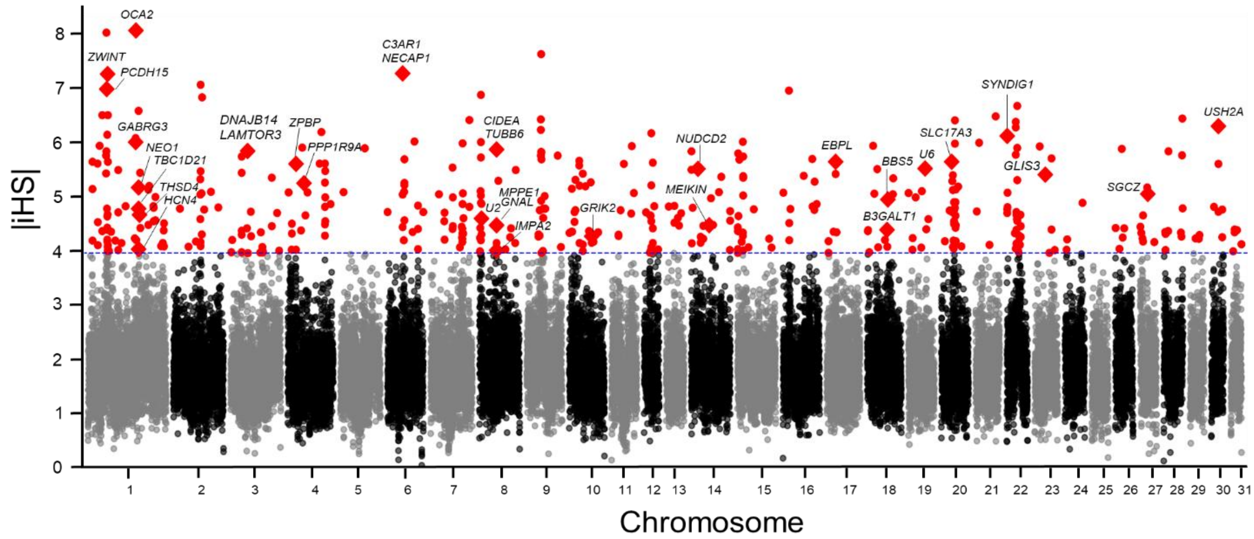

Figure S11. Genome-wide distribution of integrated haplotype score (iHS) in 57 modern Thoroughbred horses. Each point represents the iHS value calculated in sliding genomic windows (50Kb) across the autosomes (chromosomes 1–31). Grey points indicate genome-wide background variation, while red points highlight windows exceeding the significance threshold ($p < 0.01$, indicated by the horizontal dotted line), representing candidate regions under recent positive selection. Annotated genes correspond to loci within or near the most extreme iHS peaks, highlighting putative targets of selection in modern Thoroughbreds.

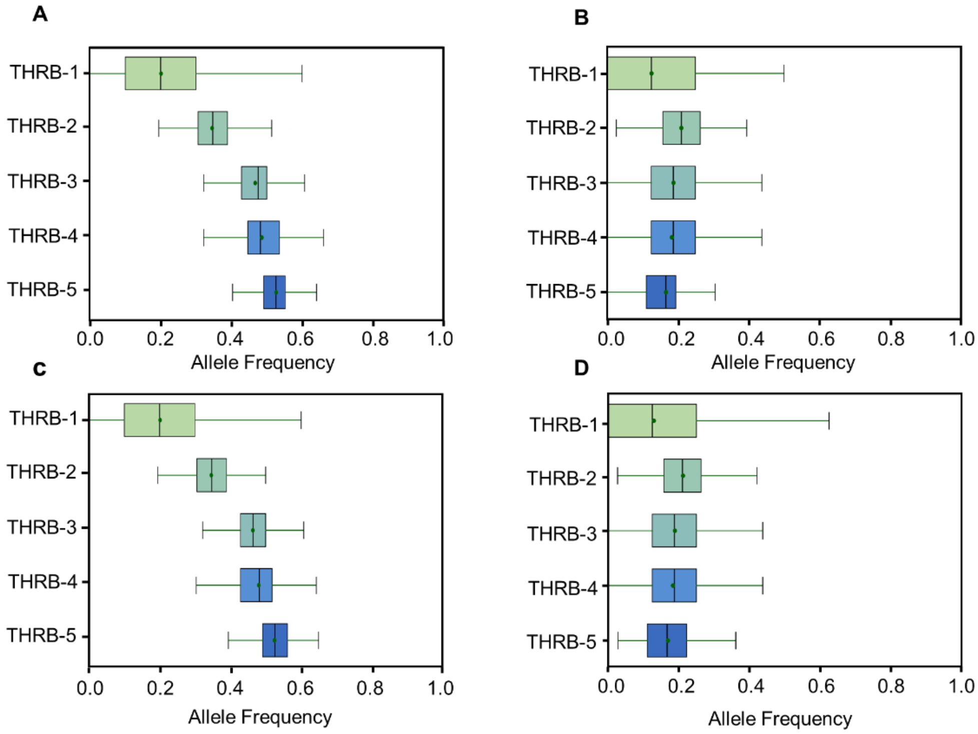

Figure S12. Allele frequency trajectories in the *MSTN* locus. (A, B) Allele frequency trajectory at position chr18:65,924,323 (rs69125012) of the *MSTN* locus. (C, D) Allele frequency trajectory at position chr18:65,983,696 (rs69125077) of the *MSTN* locus. (A, C) Allele frequencies estimated using all samples (N=170), where five individuals were randomly resampled 1,000 times per group across the same five time periods. (B, D) Allele frequencies were calculated using 1,000 replicates for a subset of individuals grouped into five time periods (N=57) (Table S9), following the procedure used in the second PBS scan. Groups: THRB-1: 1965–1969; THRB-2: 1970–1979; THRB-3: 1980–1989; THRB-4: 2000–2009; THRB-5: 2010–2020.

SUPPLEMENTARY REFERENCES

26. Alexander, D.H., Novembre, J., and Lange, K. (2009). Fast model-based estimation of ancestry in unrelated individuals. Genome Res. 19, 1655–1664.
32. Coombs, J., Letcher, B., and Nislow, K. (2012). GONe: software for estimating effective population size in species with generational overlap. Mol. Ecol. Resour. 12, 160–163.
69. Chang, C.C., Chow, C.C., Tellier, L.C., Vattikuti, S., Purcell, S.M., and Lee, J.J. (2015). Second-generation PLINK: rising to the challenge of larger and richer datasets. Gigascience 4, 7.
70. Lefort, V., Desper, R., and Gascuel, O. (2015). FastME 2.0: a comprehensive, accurate, and fast distance-based phylogeny inference program. Mol. Biol. Evol. 32, 2798–2800.
71. Patterson, N., Price, A.L., and Reich, D. (2006). Population structure and eigenanalysis. PLoS Genet. 2, e190.
72. Patterson, N., Moorjani, P., Luo, Y., Mallick, S., Rohland, N., Zhan, Y., Genschoreck, T., Webster, T., and Reich, D. (2012). Ancient admixture in human history. Genetics 192, 1065–1093.
